# Recovering new viruses from New Mexico soils

**DOI:** 10.1128/mra.00908-25

**Published:** 2025-10-16

**Authors:** Kelli Feeser, Reid Longley, La Verne Gallegos-Graves, Michaeline Albright, Migun Shakya

**Affiliations:** 1 Bioscience Division, Los Alamos National Laboratory 5112, Los Alamos, New Mexico, USA; 2 Department of Crop and Soil Sciences, North Carolina State University 242502, Raleigh, North Carolina, USA; DOE Joint Genome Institute, Berkeley, California, USA

**Keywords:** soil microbiology, phage, metagenome, high altitude, virome

## Abstract

Here, we utilized metagenomic and size-filtered virome sequencing to recover 4,157 medium, high, or complete quality viral genomes from soils taken from three high elevation sites in New Mexico, USA. Among recovered viral genomes, 90% were from size-filtered samples, indicating the importance of this enrichment in assessments of complex viromes.

## ANNOUNCEMENT

Viral communities are diverse and play important roles in soil ecosystems; however, they remain undercharacterized (1). To assess previously uncharacterized viral communities, we collected four soil samples from similar elevations at each of three different high desert mountains in New Mexico (Table 1). A total of 30 g of soil was taken for each sample and was split for processing of bulk metagenomes and size-filtered viromes (*n* = 24, 12 viromes and 12 metagenomes). Soils were initially processed by 1:1 resuspension in protein supplemented phosphate-buffered saline (PPBS) elution buffer followed by shaking, centrifugation, and size filtration. Bulk metagenomic DNA was then extracted from 550 µL of 11-µm filtrate, while viromes were processed by extracting DNA from 0.22-µm filtrate. DNA was extracted using the DNeasy PowerSoil Kits (Qiagen, USA). DNA extractions were performed using a modified protocol from (2). The exact extraction protocol is available on protocols.io. Illumina libraries were prepared following manufacturer’s instructions with the NEBNext Ultra DNA II Library Preparation Kit (New England Biolabs, USA), followed by sequencing with 151 bp paired-end reads on the Illumina NextSeq (Illumina, USA). Following sequencing, bioinformatic processing was performed with default parameters except where otherwise noted. Raw reads were quality controlled and had adapters removed using FaQCs v2.10 (3). Metagenomes were assembled using metaspades v3.12 with default parameters and k-mer lengths of 21, 33, 55, and 77 bp (4). Resulting contigs were classified to detect viruses using geNomad v1.9.0 and further checked for quality using checkV v1.0.3 (5, 6). Viruses identified as medium, high, or complete quality were retained for further analysis. Complete viral genomes were annotated with pharokka v1.7.0 (7). Viral genomes were then assessed using iPHoP v1.3.3 to predict their bacterial hosts (8). Viral sequences were clustered into species level vOTUs using *blastn* in BLAST +v2.16.0 according to the Minimum Information about an Uncultivated Virus Genome (MIUViG) specifications (9, 10).

**TABLE 1:** Sequencing and assembly results of soil metagenomes

Sample	Site	Type	Raw read no. (millions)	Metagenome size (Mb)	Contig no.	N50	%GC	Provirus no.	Complete virus no.	Elevation (m)	GPS	SRA
p1bMG	Pajarito	Metagenome	136.7	153.6	85,664	1,652	61.6	1	0	2,381	35.873548N, 106.345265W	SRR33590015
p1bMV	Pajarito	Virome	130	389.7	143,205	3,369	51.3	8	55	2,381	35.873548N, 106.345265W	SRR33590014
p2bMG	Pajarito	Metagenome	182.5	143.9	73,249	1,909	63.1	1	1	2,523	35.878239N, 106.345587W	SRR33590003
p2bMV	Pajarito	Virome	139.1	134.6	50,498	3,187	54.8	9	44	2523	35.878239N, 106.345587W	SRR33589998
p3bMG	Pajarito	Metagenome	31.1	3.8	1,766	2,118	59.4	0	1	2,678	35.887406N, 106.367832W	SRR33589997
p3bMV	Pajarito	Virome	143.6	156.2	60,752	3,028	56.6	1	41	2,678	35.887406N, 106.367832W	SRR33589996
p4bMG	Pajarito	Metagenome	190.5	7.9	4,351	1,819	61	0	2	2,852	35.894103N, 106.391385W	SRR33589995
p4bMV	Pajarito	Virome	218.2	578.7	203,091	3,657	57.6	13	173	2,852	35.894103N, 106.391385W	SRR33589994
s1bMG	Santa Fe	Metagenome	179.9	338	135,179	2,829	60.9	14	18	2,291	35.727892N, 105.843008W	SRR33589993
s1bMV	Santa Fe	Virome	156.6	362.3	137,854	3,093	61.6	8	28	2,291	35.727892N, 105.843008W	SRR33589992
s2bMG	Santa Fe	Metagenome	188.5	298.2	142,244	2,124	58.8	3	2	2,541	35.750397N, 105.82831W	SRR33590013
s2bMV	Santa Fe	Virome	185.7	452.5	201,892	2,369	58.5	4	20	2,541	35.750397N, 105.82831W	SRR33590012
s3bMG	Santa Fe	Metagenome	175.1	112	59,613	1,850	59.6	0	2	2,798	35.779612N, 105.810679W	SRR33590011
s3bMV	Santa Fe	Virome	117.2	286.9	130,221	2,316	59.8	2	6	2,798	35.779612N, 105.810679W	SRR33590010
s4bMG	Santa Fe	Metagenome	144.8	12.7	5,023	2,494	59.9	0	0	2,950	35.793646N, 105.800465W	SRR33590009
s4bMV	Santa Fe	Virome	273.9	458.5	218,828	2,154	60.2	1	5	2,950	35.793646N, 105.800465W	SRR33590008
t1aMG	Taos	Metagenome	151.3	248.7	132,208	1,736	64.2	0	1	2,285	36.53568N, 105.561685W	SRR33590007
t1aMV	Taos	Virome	146.1	246.9	127,628	1,903	60.2	2	4	2,285	36.53568N, 105.561685W	SRR33590006
t2bMG	Taos	Metagenome	374.9	379.1	188,924	1,966	64	1	0	2,578	36.5841N, 105.496496W	SRR33590005
t2bMV	Taos	Virome	174.2	643.6	280,575	2,430	59.6	14	34	2,578	36.5841N, 105.496496W	SRR33590004
t3aMG	Taos	Metagenome	222.5	344.4	154,390	2,273	55.9	8	16	2,818	36.596973N, 105.448809W	SRR33590002
t3aMV	Taos	Virome	154.9	475.9	179,380	3,244	57.9	17	27	2,818	36.596973N, 105.448809W	SRR33590001
t4aMG	Taos	Metagenome	168.6	1,046.6	387,412	3,299	56.4	13	16	3,015	36.578601N, 105.438383W	SRR33590000
t4aMV	Taos	Virome	128	796.6	273,208	3,872	56	20	67	3,015	36.578601N, 105.438383W	SRR33589999

**Fig 1:**
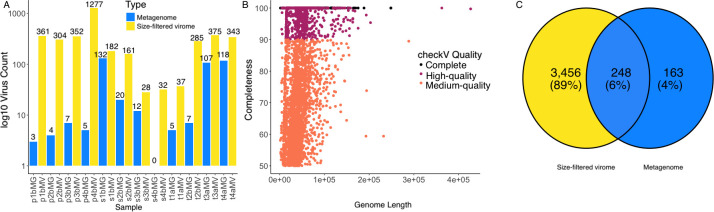
(**A**) Size selection increases recovery of viruses from soil samples compared to metagenomes. (**B**) A total of 4,157 recovered viral genomes vary in quality and length metrics. (**C**) Distribution of VOTU clusters from metagenome vs size-filtered virome samples indicates a high number of unique viruses from size-filtered virome samples.

Assembly sizes ranged between 3.8 Mb and 1,046.6 Mb (Table 1). From these assemblies, we recovered 4,157 viruses of medium, high, or complete quality. Filtered viromes consistently recovered higher numbers of viruses (average = 311) compared with metagenomes (average = 35) (Fig. 1A). Among the recovered viruses, 563 were identified as being complete, 995 were high quality, and 2,599 were medium quality (Fig. 1B). Clustering of the 4,157 viral genomes into species level vOTUs created 3,867 clusters, indicating that the majority of recovered viral genomes were unique. The majority (89%) of clusters were composed of viruses recovered only from viromes, indicating that size-filtered samples produced maximum diversity (Fig. 1C). Host analyses using iPHoP identified 124 complete or high-quality viruses, which could be assigned to a host with >90% confidence. Phage sequences were associated with common soil bacterial genera, including *Mycobacterium*, *Pseudomonas*, and *Streptomyces*. Our results agree with previous studies, indicating that size filtration-based viral enrichment methods are a valuable tool to recover viral genomes from complex communities including soil (11, 12). We expect that this data set will act as a valuable reference as the diversity of viruses in soil continues to be uncovered.

## Data Availability

The raw reads have been deposited to NCBI SRA (Bioproject no. PRJNA1263537), and complete viral genomes have been deposited to GenBank (accession no. PV944424-PV944986). Medium and high-quality viral genomes are available on Dryad (https://doi.org/10.5061/dryad.h1893200p).

## References

[1] Graham EB, Camargo AP, Wu R, Neches RY, Nolan M, Paez-Espino D, Kyrpides NC, Jansson JK, McDermott JE, Hofmockel KS, Soil Virosphere Consortium. 2024. A global atlas of soil viruses reveals unexplored biodiversity and potential biogeochemical impacts. Nat Microbiol 9:1873–1883. doi:10.1038/s41564-024-01686-x 38902374 PMC11222151

[2] Albright MBN, Gallegos-Graves LV, Feeser KL, Montoya K, Emerson JB, Shakya M, Dunbar J. 2022. Experimental evidence for the impact of soil viruses on carbon cycling during surface plant litter decomposition. ISME Commun 2:24. doi:10.1038/s43705-022-00109-4 37938672 PMC9723558

[3] Lo CC, Chain PSG. 2014. Rapid evaluation and quality control of next generation sequencing data with FaQCs. BMC Bioinformatics 15:366. doi:10.1186/s12859-014-0366-2 25408143 PMC4246454

[4] Nurk S, Meleshko D, Korobeynikov A, Pevzner PA. 2017. metaSPAdes: a new versatile metagenomic assembler. Genome Res 27:824–834. doi:10.1101/gr.213959.116 28298430 PMC5411777

[5] Camargo AP, Roux S, Schulz F, Babinski M, Xu Y, Hu B, Chain PSG, Nayfach S, Kyrpides NC. 2024. Identification of mobile genetic elements with geNomad. Nat Biotechnol 42:1303–1312. doi:10.1038/s41587-023-01953-y 37735266 PMC11324519

[6] Nayfach S, Camargo AP, Schulz F, Eloe-Fadrosh E, Roux S, Kyrpides NC. 2021. CheckV assesses the quality and completeness of metagenome-assembled viral genomes. Nat Biotechnol 39:578–585. doi:10.1038/s41587-020-00774-7 33349699 PMC8116208

[7] Bouras G, Nepal R, Houtak G, Psaltis AJ, Wormald PJ, Vreugde S 2. 2023. Pharokka: a fast scalable bacteriophage annotation tool. Bioinformatics 39. doi:10.1093/bioinformatics/btac776 PMC9805569 36453861

[8] Roux S, Camargo AP, Coutinho FH, Dabdoub SM, Dutilh BE, Nayfach S, Tritt A. 2023. iPHoP: An integrated machine learning framework to maximize host prediction for metagenome-derived viruses of archaea and bacteria. PLoS Biol 21:e3002083. doi:10.1371/journal.pbio.3002083 37083735 PMC10155999

[9] Camacho C, Coulouris G, Avagyan V, Ma N, Papadopoulos J, Bealer K, Madden TL. 2009. BLAST+: architecture and applications. BMC Bioinformatics 10:421. doi:10.1186/1471-2105-10-421 20003500 PMC2803857

[10] Roux S, Adriaenssens EM, Dutilh BE, Koonin EV, Kropinski AM, Krupovic M, Kuhn JH, Lavigne R, Brister JR, Varsani A, et al.. 2019. Minimum information about an uncultivated virus genome (MIUViG). Nat Biotechnol 37:29–37. doi:10.1038/nbt.4306 30556814 PMC6871006

[11] Göller PC, Haro-Moreno JM, Rodriguez-Valera F, Loessner MJ, Gómez-Sanz E. 2020. Uncovering a hidden diversity: optimized protocols for the extraction of dsDNA bacteriophages from soil. Microbiome 8:17. doi:10.1186/s40168-020-0795-2 32046783 PMC7014677

[12] Santos-Medellin C, Zinke LA, Ter Horst AM, Gelardi DL, Parikh SJ, Emerson JB. 2021. Viromes outperform total metagenomes in revealing the spatiotemporal patterns of agricultural soil viral communities. ISME J 15:1956–1970. doi:10.1038/s41396-021-00897-y 33612831 PMC8245658

